# Education and debate: a manifesto for ethics and values at annual healthcare conferences

**DOI:** 10.1080/17571472.2016.1244152

**Published:** 2016-10-24

**Authors:** Andrew Papanikitas

**Affiliations:** ^a^ NIHR Academic Clinical Lecturer in General Practice, Department of Primary Care Health Sciences, University of Oxford, Oxford, UK

**Keywords:** Ethics, professional, healthcare, conference

## Abstract

In this paper I discuss the ways in which the conference stream ethics and values manifested at the 2015 RCGP Annual Conference in Glasgow, and the ways in which it is planned for the 2016 RCGP Annual Conference in Harrogate. The 2015 RCGP had plenaries, oral presentations, breakout symposia, a debate, and a poster stream. I briefly discuss each in turn before offering a manifesto (a public statement of aims and proposed policy) for ethics and values at healthcare conferences. It is my hope that others will critique this, flesh it out further and even consider how ethics and values relate to conferences for healthcare workers of various specialities. A conference provides opportunities for ethics and values discussion that are potentially distinctive from any other kind of forum. Because conferences offer the potential for knowledge and attitudes to be revisited and revised, issues can be ‘unsettled’ in a way that permits different perspectives to be more fully discussed.

## Why this matters to me

As a general practitioner who teaches ethics and law to general practitioners (GPs) in the UK and who has conducted qualitative research on ethics education for GPs, I am aware that there are relatively few opportunities or safe forums to discuss ethics, values and their ramifications in practice, with colleagues. These safe forums are often associated with formal educational settings such as a half-day of classroom teaching in a GP training scheme or some dedicated time in a GP trainers’ group. The value of such discussions may be limited by formal assessment criteria or absent opportunities for individuals to influence professional consensus (GP trainees, for example may be more interested in demonstrating competencies for practice rather than changing the world). Discussion at a healthcare conference offers expanded opportunity for shared learning and a setting where matters can be discussed in ways that are productively unsettled, because healthcare conferences showcase new knowledge and generate new consensuses.

## Key messages

• Medical, healthcare and/or biomedical ethics conferences attract educators and practitioners as well as academics, but the importance of healthcare conferences as sites for ethical discussion should not be overlooked
• Participants at the 2015 RCGP Annual Conference expressed interest in discussing ethics and values
• Ethical forums (at conferences and elsewhere) need to be continually facilitated if they are to be used.
• A conference stream ought to be open to multiple ways of expressing the ethics and philosophy of healthcare, contextualized to the conference faculty and delegates

## Introduction

In this paper I discuss the ways in which the conference stream ‘ethics and values’ manifested at the 2015 RCGP Annual Conference in Glasgow and is planned for the 2016 RCGP Annual Conference in Harrogate. The 2015 conference boasted plenaries, oral presentations, breakout symposia, a debate, and a poster stream. The 2016 conference will include a poster stream and a fringe event on the ethics of flourishing and survival. I briefly discuss each in turn before offering a manifesto (a public statement of aims and proposed policy) for ethics and values at healthcare conferences. It is my hope that others will critique this, flesh it out further and even consider the role of conferences in postgraduate ethics and healthcare education more broadly.

## Issues raised in the plenary lectures

At the 2015 RCGP Annual Conference, plenary sessions had a strong moral flavour: Human rights lawyer Shami Chakrabarti discussed the importance of human rights and civil liberties [1] and reminded the delegates that healthcare professionals constituted a politically influential group, often involved in exposing human rights abuses in the UK and abroad. Talking about why he wrote his 1978 bestselling medical novel, ‘The House of God.’ Samuel Shem (aka Professor Stephen Bergman from New York University) discussed the threat of healthcare workers’ disconnection from each-other and from vocation itself.[2] Shem controversially talked about how his generation of medical students publically protested against the Vietnam War and suggested that if doctors felt that a contract was not fair or safe, they ought to strike. Crimewatch presenter, Nick Ross suggested that crime should be treated epidemiologically, like a disease.[3] Professor Frede Olesen from Aarhus University offered biologically and empirically founded arguments for the importance of good clinician–patient relationship. These plenaries expose delegates to discussions that are taking place in society that clearly might influence but might not explicitly feature in the day to activities of a healthcare worker. They also offer delegates the potential to respond to what they hear, by questioning the speaker, by recognising reasons to engage with an issue (for example through political action) or by discussion with colleagues how to make the best of a changing world (links to two of the talks at the conference and a similar talk by Samuel Shem are listed below).

## Videos podcasts linked to the above plenaries

Professor Frede Olesen from Aarhus University discussing the healing power of the doctor-patient relationship – in biomedical terms. *See video:* https://www.youtube.com/watch?v=bpGvHZfBGd8

Shami Chakrabarti discussing the importance of legal protection of human rights and resulting civil liberties. *See video:* https://www.youtube.com/watch?v=bpGvHZfBGd8

Samuel Shem aka Professor Stephen Bergman from New York University discussed why he wrote, ‘the House of God’ and human disconnection in healthcare as a source of distress. http://torch.ox.ac.uk/interview-professor-stephen-bergman

## Breakout sessions

Three breakout sessions which explicitly concerned the ethics and philosophy of healthcare practice all filled their conference venues (these are each briefly described below).

The RCGP Committee on Medical Ethics’ debate asked ‘Who knows best – the patient or the clinician?’ This highlighted the difficulty in defining honest and meaningful patient-centred choice. Chaired by Professor Simon Gregory (RCGP Committee on Medical Ethics), two professors of medical law, Hazel Biggs from Southampton and Charles Foster from Oxford depolarised the debate by arguing that there is space for professional expertise and respect for patient autonomy. They discussed the issue that patient choices are influenced by a number of factors, making ‘true’ autonomy quite a difficult concept with implications for clinical practice. Debate was more polarised among the audience with points such as the distinction between needs and desires and limitations on medical influence being usefully rehearsed. Conversations about meaningful patient choice and shared decision-making continued beyond the session. Previous ethics debates at the conference have included thorny issue such as how to deal with financial conflicts of interests such as sponsored education and unpacked the issues implicit in shared electronic patient healthcare records.[4]

Unfortunately the debate ‘Who knows best – the patient or the clinician?’ was concurrent with a symposium on ‘Flourishing practice’, forcing the delegates to choose. The symposium was led by Peter Toon and in connection with his new book.[5] Peter Toon and assembled delegates asked what the virtues and internal goods of practice might be. Internal goods include things like mastery of a skill and distinguished from external goods such as status or money. They highlighted many of the reasons why GPs do not feel that they are flourishing – such as lack of time and work pressures squeezing out opportunity for reflection.

The, ‘Inside GP ethics’ workshop began with brief presentations from the Chair of the ethics committee (Simon Gregory), a senior educator involved in teaching ethics (John Spicer), and a GP-philosopher whose emphasis has been on virtue ethics (Peter Toon). The discussion included issues such as ethical aspects of the trainee in difficulty and broader discussion about the place of philosophy in clinical thinking. The brief presentations offered delegates a taste of what thinking was being done in their professional organisations and educational bodies, and what relevant support for clinicians those organisations were offering – including the work of the RCGP and postgraduate education organisations such as Health Education England). The discussion offered an opportunity for the RCGP Ethics Committee and educators in the room to hear learn about the issues affecting delegates in their clinical, educational and managerial roles.

## The 2015 poster stream

The call for posters elicited contributions including: empirical work on patient choice regarding place of death (the winning poster in the category), the ethical puzzle of clinician self-care, and issues for clinical practise arising from ethnic diversity. A fun poster with a serious message applied UK General Medical Council ethical standards to doctors in the television science-fiction programme Star Trek. Public expectation of the medical profession is potentially shaped by the characterisation of doctors in popular media. Many of the Star Trek doctors behaved in very paternalistic ways – The authors noted that most ethical doctor in the programme was not human but an artificial intelligence, who developed person-centred skills with time and experience. At least one of the posters, which asked whether GPs should avoid making ethical decisions was subsequently published in the British Journal of General Practice.[6]

## The future – 2016 and beyond

There is clearly appetite among practitioners for ethical discussion and debate at the RCGP conference. This has the potential to improve clinician welfare and decision-making though making resources available when clinicians face a dilemma. The RCGP Conference offers a protected space where issues can be considered at a remove from clinical pressures (for example, the time delegates spend at a conference is generally protected from demands to attend to immediate patient needs or workplace administration), with the luxuries of time and available expertise. The ‘Energising primary care’ conference at Harrogate will have a poster section for ethics and values and a fringe meeting to discuss an topic of ethical moment: The ethics of survival and the ethics of flourishing. There has been much talk about resilience and survival, and hard choices, rhetorical gaps and going extra miles in general practice. Statements about how to generate excellence in primary healthcare coexist alongside open letters from overwhelmed practitioners contemplating early retirement. Flourishing, virtue and excellence can seem like aspirations of the ivory towers of academia rather the swampy lowlands of practice. At worst, rhetoric of excellence can seem like a recipe for moral failure. And yet general practice and primary care need energising –this includes a philosophy of practice whatever that might be. Members of the RCGP ethics committee and invited panel members will host an open discussion on the ethics of everyday general practice. We will ask: Should we concentrate on surviving or flourishing? Ought we to think about both? What are the ways that that such ideas are helpful or unhelpful in practice? How can we best energise the ethics of general practice and primary healthcare in the 21st century? This fringe meeting is intended as an open discussion conducted under Chatham House rule (A rule of anonymity where delegates are free to discuss the content of a meeting but without naming colleagues or their institutions. In theory this allows a more frank discussion that is illustrated by real experiences).

## From is to ‘ought’

An ethics and values stream at a primary care (indeed any general healthcare conference) should comprise:

(1) Representation on the conference management committee. This can serve at least two key purposes: to represent relevant interests to the conference committee and to represent the conference to groups that might participate. In 2015 the representative was drawn from the organisation’s own ethics committee. The expectations of representative to be a conduit and not just to tout their own interests should be clear.
(2) A poster theme: these might include (among other things) reports of other events, posters raising issues, posters displaying empirical research and more light-hearted posters using the arts and popular to make serious points about healthcare.
(3) Oral papers: A key issue is whether to embed ethics and values papers in other streams or have an ethics and values stream. Embedding a presentation on ethics and education for example in education rather than in ethics arguably means that the presenter primarily interacts with educators.
(4) The Ethics Committee Debate: This was attended by a full room at the RCGP conference in 2015. Most professional groups have an ethics committee that filters issues of societal and professional moment. This is a resource for both selecting an issue for discussion and sourcing good speakers.
(5) An ‘Ethics at the frontline’ session: in 2015 the RCGP ethics committee led such a session and drew a full lecture room. This offers delegates and faculty the opportunity to start a conversation. Ethics committee members should attend such a meeting, which should be a safe space conducted under Chatham House rule (see above) and an opportunity to horizon-scan for ethical issues in practice.
(6) Other opportunities to share learning and raise issues should be highlighted. Louhiala et al. have maintained an online ethics support network which reports back to and is the focus of a symposium at the Finnish Medical Association annual conference.[7] Dunn et al. developed an online interactive casebook for Singaporean clinicians that has been used and adapted in several other countries.[8] There are also informal social media networks such as the Primary Care Ethics LinkedIn Group – an intermittently active community of about 400 academics, educators, policymakers stakeholders and clinicians.[9]

## Concluding reflections

This journal has previously published reports of conferences dedicated to the ethical issues arising in primary healthcare.[10,11] By contrast, In this paper, I have described some of the activities and intellectual content of the 2015 RCGP Annual conference. This is to illustrate the distinctive way in which ethics and values can be a part of such an event and a distinctive way in which a healthcare conference can be a space for the discussion of ethics and values. A conference generates issues and discussions of current and future ethical importance. It draws together people with shared aims and people who have a variety of perspectives. The time-out-of-practice aspect can enable discussion and reflection, which might only otherwise be possible online. It can bring changes in society to the attention of healthcare workers, educators and leaders. It can enhance the moral agency of individuals in any given profession and society by reminding them of the ways in which they can engage with politics. It can give individuals the opportunity to put their concerns directly to educators, professional leaders and policymakers. As well as ‘how should we respond to this situation?’ question, ‘how can we change this situation?’ becomes as reasonable question for discussion. Shared learning and conversations at a conference are possible in ways that cannot happen online, in a classroom session or in a Balint group. When a conference offers new knowledge, a forum for discussing how delegates ought to behave in their professional lives and ways in which delegates might influence the society in which they practice, it usefully unsettles what we know about ‘is’ and ‘ought’.

## Disclosure statement

The author is part of an informal research and teaching network that aims to study and teach postgraduate healthcare and primary healthcare ethics.

## Governance

The author is writing in his capacity as an academic clinical lecturer at the University of Oxford and a medical ethics teacher for several organisations and not on behalf of the RCGP Committee on Medical Ethics. RCGP England or RCGP London
